# Designing health care inclusively for people with low incomes: A policy-focused evidence brief

**DOI:** 10.1016/j.puhip.2026.100768

**Published:** 2026-03-24

**Authors:** Adnaan Ghanchi, Anna Gkiouleka, Danielle Lamb, Liam Loftus, Payam Torabi, John Ford

**Affiliations:** a Department of Public Health and Primary Care, University of Cambridge, UK; b Wolfson Institute for Population Health, Queen Mary University of London, UK; c NIHR Applied Research Collaboration, UCL, UK; d The Aberfeldy Practice, UK

**Keywords:** Health inequalities, Low-income populations, Healthcare access

## Abstract

**The policy challenge:**

Healthcare systems often fail to meet the needs of low-income populations, reinforcing disparities in access, affordability, and quality of care. These inequities are not incidental but the result of design choices that prioritise efficiency and standardisation over inclusivity. Even in high-income countries with publicly funded healthcare systems, hidden costs, transport, parking, lost income, childcare, and the financial burden of following medical advice, can prevent people from accessing care. These financial barriers drive delays in treatment, missed appointments, and poorer health outcomes, entrenching health inequities.

**Key evidence to inform policy:**

This brief synthesises evidence from systematic and realist reviews, randomised controlled trials, qualitative studies, and programme evaluations, prioritising the most robust and policy-relevant findings of the structural barriers that low-income populations face in high-income healthcare systems. It identifies three challenges: (1) the cost of time and travel, (2) the lack of financial hardship awareness in healthcare settings, and (3) the affordability of care beyond direct medical costs. Solutions exist. Flexible appointment models, including evening and remote consultations, can mitigate financial strain. Community outreach services, such as mobile health units and pop-up clinics, improve engagement with preventive care. Social needs screening, widely used in North America, has been shown to improve health outcomes by identifying financial hardship and linking patients to support services. Food prescription programs offer an emerging model for addressing health and nutrition insecurity.

**Further considerations and implications:**

While healthcare alone cannot eliminate poverty, it can ensure that low-income populations are not left behind. Policymakers should embed equity into healthcare design, integrating financial support programs, expanding outreach initiatives and removing hidden costs that act as barriers to care.

## Highlights

• Key barriers for low-income populations include time, travel costs and financial hardship awareness.
• Hidden costs like lost wages, childcare and transport, delay treatment and lead to worse health outcomes.
• Flexible appointment models, mobile health units, and outreach services improve access for low-income populations.
• Social needs screening and food prescription programs identify hardship and link patients to services.

## 1 Current policy challenges

**Fig. 1:**
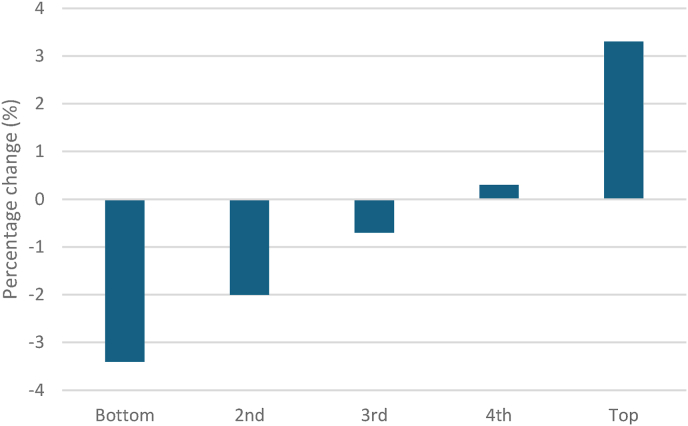
Changes in disposable household income across income distribution from 2020/21 to 2021/22 (real terms).

**Fig. 2:**
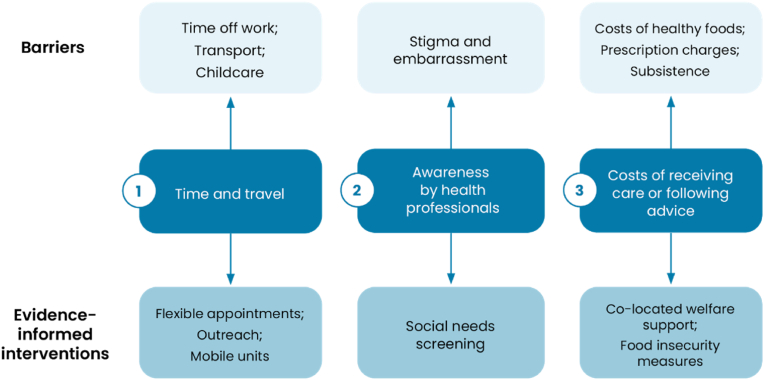
Key themes of Rapid Evidence Review.

The evidence for the association of low income and poor health is both overwhelming and well-established [1]. There are currently 14.3million people living in relative poverty in the UK(21% of the population) [2]. The mean disposable income in the UK has only increased slightly in real terms in the past 14 years, from £38,670 in 2007/08 to £39,328 in 2021/22 [3]. Income inequalities remain high; in 2022/23 a couple without children in the lowest 10% income band had a mean disposable income of £300 per week before housing costs, compared to £1200 for those in the highest 10% [4]. There are also signs that inequalities in disposable income have been worsening. Between 2020/21 and 2021/22, people in the top 20% saw their disposable income increase by 3.3%, but those in the lowest 20% saw it reduce by 3.4% [3, Fig. 1]. Globally, the last two decades have shown a gradual increase in income inequality, this includes developed countries such as the USA, Canada, Australia and European countries such as Germany and Italy [5] (see Fig. 2).

People on low incomes are more likely to have concomitant risk factors, such as smoking, poor diet, and limited physical activity. These are compounded by the fact poorer people are more likely to suffer multiple health issues, culminating in shorter lives [6]. The causal pathway is multifactorial and likely to build over the life course with factors including stress, poor quality housing, lack of access to green space, pollution, and access to cheaper ultra-processed food [7].

Addressing the root causes of income inequality lies outside the remit of the health care system. However, people who are on a low income can have problems accessing and using healthcare. In the UK, whilst NHS care is free at the point of use, there are often many hidden out-of-pocket expenses which patients must cover, such as travel, lost employment, childcare, and subsistence during unplanned attendances. These barriers may mean delay in seeking care or missed appointments or prescriptions. The NHS has a Low-Income scheme, but uptake is patchy. A qualitative study in the North East of England published in 2024, found that people on low incomes were often not aware of the financial support available and often found out “by chance”(11).

Here we review the evidence of what works to mitigate the barriers faced by people on low incomes when using health care services, while acknowledging that the root causes lie outside the responsibility of health care.

## 2 Approach to collating evidence

To identify relevant policy-focused evidence, we identified relevant and robust studies which focused on interventions or programmes that support people on low incomes. Relevant studies were judged to be action-focused to inform policy and robustness was an assessment by the authors of the data which contributed to the relevant findings. For example, only a subsection of a study may be relevant to low income patients and only this section of text was assessed for robustness. The majority of evidence was drawn from the UK, with select studies from the United States and other high-income countries where interventions were considered relevant to universal healthcare contexts.

The purpose was to gather sufficient relevant and robust evidence to draw evidence-informed recommendations for policy makers, rather than collate all the evidence on a broad policy area which would be impractical. We identified studies through [1]: Living Evidence Maps developed by the Health Equity Evidence Centre [2,8] a search of an electronic database (MEDLINE) examining what works to support people on low incomes benefit from health care, and [6]; snowballing by citation tracking and a machine learning tool (Litmaps). In total, we prioritised 21 research studies which provided the highest quality evidence on what works to ensure health care services support people on low incomes. These studies were either recent high-quality systematic reviews or particularly relevant to the policy context in countries with publicly funded universal health care provision.

## 3 Key evidence to inform policy

There are three main areas for policymakers to take action to support people on low-incomes [1]: costs associated with time and travel [2], awareness of financial difficulties by health professionals, and [6] the costs of receiving care or following advice.

## 4 Time, access and travel barriers

Problems for people on low incomes accessing health care have been described in the literature [[9], [10], [11], [12], [13]]. In a qualitative study of 24 parents and 8 Voluntary Community Social Enterprise sector staff based in the north-east of England undertaken in 2021/22, Bidmead and colleagues highlighted key issues low-income parents face [14]. This included difficulties with internet access to book appointments, the cost of remaining on hold while waiting to speak to a receptionist, the ability to take time off work to attend appointments, and the cost of childcare to attend appointments. The authors found that travel costs to primary care providers were often minimal, but attending secondary care often required multiple bus journeys or excessive parking fees, with one parent reported spending up to £50 on travel to a hospital appointment, increasing her likelihood of cancelling the appointment.

In a 2016 UK survey by Verhoef and colleagues of 574 women attending antenatal screening, 36% of women took unpaid absence to attend. 71% of women attended screening with someone else who had taken time off work [15]. The authors estimated that the time-cost to attend an appointment was £22 per visit. 10% of women reported losing income through attending the clinic, ranging widely from £3 to £250.

Ford and colleagues (2016) reviewed 163 studies examining the problems older people in rural areas on low incomes face accessing primary care in a realist review [9]. They found that an individual's financial resources impact their decision to seek help from their GP either because they were concerned about the financial implications of receiving a diagnosis or the cost of transport. Transport was especially important for older people who did not have access to a car within their household and for whom services were not nearby. While the majority managed to secure transport, their decisions often involved a trade-off between the significant effort and expense required and the perceived benefits of doing so. Younger people also report being affected by cost barriers. A UK survey of 203 young people aged 18-25 were asked about barriers in accessing mental health services; 26% of people reported cost as a major barrier [11].

Gkiouleka and colleagues undertook a realist review of interventions to address inequalities in primary care [16]. Based on the 159 studies that were included, the authors found that flexibility was one of five key guiding principles to reduce inequalities – i.e., organisations should make allowances according to different patients' needs. This would include being flexible about the model of appointment (e.g., remote or face-to-face) and timing (e.g., options outside working hours). This is supported by Tierney and colleagues’ review (2023) of telemedicine for low-income patients [17]. Based on 45 studies from the US, the authors found that for some patients, telemedicine was more affordable and increased access for those with prohibitive travel or childcare costs.

Outreach interventions with opportunistic services may overcome the costs people on low incomes face to attend healthcare. Roberts and de Souza (2016) reviewed outreach venues to increase the uptake of NHS health checks in people living in socio-economically disadvantaged areas, men, and South Asians in Buckinghamshire [18]. Based on 3849 health checks undertaken, the authors found that supermarkets and libraries had the highest uptake, but mosques and bus stations had the highest uptake for disadvantaged communities. A greater number of men also took part when services were based in manufacturing workplaces and at football matches.

Mobile drop-in stop smoking services have also been found to be effective in increasing the uptake of disadvantaged groups. Venn and colleagues (2014) evaluated drop-in stop smoking services in various public venues across Nottingham and compared them with the standard services at a fixed location [19]. Based on 811 people using the mobile service, compared to 1856 using the standard service, the authors found that people who used the mobile service were statistically significantly more likely to be from routine or manual occupational groups (33.3% vs 27.2%), first-time users of the service (67.8% vs 59.3%), and live in low-income areas with high social housing (27% vs 26%).

## 5 Awareness of issues by health professionals

Approximately 1 in 5 GP consultations are due to a social issue within the UK, costing approximately £400 million per year [20]. Most of these consultations involved issues directly related to poverty such as housing (77%) and work/unemployment (76%) and could be handled by more appropriate staff. Bidmead and colleagues’ qualitative study in the north-east of England found a lack of a systemic approach to supporting patients living in poverty, with help usually reliant on individual health professionals [14]. A survey of 526 people with mental health problems by Mind found that people feel significant shame and stigma if they do not have enough money, especially if there are visible issues, such as being on benefits, using food banks, being in debt, or having to ask for help from friends or family [21].

Healthcare organisations in North America have been “screening” for social needs for several years. Social needs screening involves asking patients about social issues in order to identify those who may benefit from help and refer them to onward support. Yan and colleagues (2022) undertook a review of social needs screening in clinical settings [22]. Half of the 28 included studies were RCTs and 11 reported on health outcomes. Interventions ranged from staff identifying social risk and distributing a leaflet of community resources or signposting to support from a patient navigator or social worker. The authors found that positive short-term impacts included increased smoking cessation rates, improved child health (caregiver self-report), better blood pressure control, decreased intimate partner violence, lower cholesterol, increased fruit and vegetable consumption, and improved self-rated health. The authors also found evidence for improved adherence to treatment, immunisation rates, reduced A&E attendance and hospital readmissions. Other reviews have found that social needs screening is successful in identifying people with financial problems [[23], [24], [25]]. De Marchis and colleagues (2023) reviewed implementation factors of social needs screening and found that time was the most cited barrier, but that standardisation of tools and workflow helped [26]. The authors also found that community, health workers and technology helped patients to share information and facilitated screening in resource-limited settings.

Moscrop and colleagues reviewed the reasons for and against social needs screening in 2019(26). Most academic articles supported social needs screening to improve outcomes, support health care service monitoring and provision and population health approach. Eight of 138 articles listed raised concerns about potential harms including professional boundaries and onward use of data.

## 6 Costs of receiving care or following advice

Patients on low incomes can also find services which require copayment, such as prescription charges, difficult and costs during unplanned hospital attendance or to follow the advice of healthcare staff.

In England, there is a prescription charge currently of £9.90 for people aged 18-60 years. There are several exemptions, including those who receive means-tested benefits, certain conditions, pregnant women, and those who have recently had a baby. Both Scotland and Wales have abolished the prescription charge. An evaluation in 2018 in Scotland found mixed results and could not draw conclusions on the impact on health inequalities [27]. An evaluation in Wales in 2011 found a statistically significant increase in the number of prescriptions and a reduction in the number of medications bought. However there was little or no impact on those on the lowest incomes [28].

Bidmead and colleagues in the qualitative study also reported the cost of food and drinks during hospital attendance as significant challenges [14]. They reported that food was not provided for parents staying with a child, even if the mother is breastfeeding. It was particularly costly for unplanned admissions when parents and carers could not plan ahead. The authors also found costs associated with discharge from ED or an admission, especially when this was with children or when public transport was not operating.

There can be ongoing financial consequences of illness. Ngan and colleagues (2022) looked at the financial implications of surviving cancer in the UK(37). Based on 29 included studies, the authors found that many survivors and/or carers faced severe financial problems, such as debt and difficulty paying a mortgage, leading to mental health problems and being forced to return to work prematurely.

Several studies reported on the cost of eating a healthy diet, especially when advised by their doctor because of a health problem. The Food Foundation estimate that most deprived fifth of the population would need to spend 50% of their disposable income on food to meet the cost of the Government recommended healthy diet [29]. This compares to just 11% for the least deprived fifth. Woodward and colleagues (2024) found 12 studies that reported that people with diabetes on low incomes found it difficult to afford healthy food [30]. Marteau and colleagues noted that increasing household income in poorest households increases spending on fruit and vegetables and reduces spending on tobacco and alcohol [31].

Two reviews examined the colocation of welfare advisors in health care settings. Reece and colleagues (2022) reviewed 14 studies of co-located welfare services in the UK, mostly in general practice through Citizen's Advice and reaching people on low incomes and those not in work [32]. The authors found that all studies found an improvement in financial security with an average financial gain of £776 to £3656 and an improvement in the financial literacy of both patients and staff. There was also an 7% average reduction in GP attendance after co-location of a welfare advisor. Young and colleagues (2022) included 15 articles which evaluated free to access advice services on social welfare issues [33]. The authors found that improvements in mental health and wellbeing services and co-locating services supported collaboration to tackle the social determinants of health.

Several Food is Medicine initiatives across the US support patients to eat healthily, addressing food insecurity and nutrition simultaneously. Mozaffarian and colleagues (2024) in their summary of the programme highlight treatment support patients can receive from food prescriptions, medically tailored groceries, and medically tailored meals [34]. Little and colleagues (2022) reviewed 23 studies examining food prescription programmes [35]. The authors found food prescriptions improved fruit and vegetable consumption and reduced food insecurity, but there remained barriers of stigma, transport, and nutritional literacy. Hager and colleagues (2023) evaluated 22 food prescription sites across 12 US states, including 3881 patients from low-income areas [36]. The researchers found, at 6 months, a reduction in food insecurity, improved self-reported health, reduced HbA1c, blood pressure, and obesity. However the transferability of this to a universal, publicly-funded health care context is unknown with the Institute for Health Equity recommending cash transfers rather than food aid [37].

## 7 Limitations

Much of the available research is based on high-income countries, such as the USA particularly those without universal healthcare, making it difficult to generalize findings to diverse global health systems. While some interventions, such as social needs screening and co-located welfare services, show promise, their effectiveness varies depending on healthcare infrastructure, economic conditions, and policy environments.

Additionally, each intervention reviewed is highly specific to its cultural and economic context at the time of study. Healthcare access is shaped by national policies economic conditions, welfare systems, and societal attitudes toward poverty, meaning that an intervention effective in one setting may not necessarily be transferable to another. Finally, disparities in data collection across studies and countries further limit cross-national comparisons. More research is needed to assess how healthcare interventions interact with broader social and economic structures over time and to identify scalable solutions that can be adapted to different policy environments.

## 8 Further considerations and implications

This review highlights the financial barriers that low-income populations face in accessing healthcare, but also demonstrates that evidence-based solutions exist - though none address the root cause. Flexible appointment models and telemedicine increase access for patients facing prohibitive travel or childcare costs. Social needs screening has improved blood pressure control, smoking cessation, and fruit and vegetable consumption, while reducing A&E attendance and hospital readmissions. Co-located welfare services have delivered average financial gains of £776–£3656 per patient and a 7% reduction in GP attendance. Outreach services in community venues have increased uptake of preventive care among disadvantaged groups. These approaches should be embedded into routine healthcare design. Ultimately, the most effective way to improve healthcare for low-income populations is to increase their incomes. Higher earnings enable people to afford healthier food, better housing, and reduce financial stress - key factors that influence long-term health. Policymakers designing healthcare strategies must recognise that while targeted interventions can reduce immediate barriers, meaningful progress requires economic policies that lift the poorest out of poverty, ensuring a fairer distribution of health resources and better overall health outcomes.

**Case Study: Poverty Proofing in North East England**

Children North East is a charity in the North East of England that delivers services, support, and initiatives for children, young people, and families. They have developed a Poverty Proofing stream to think about how to ensure healthcare services do not exclude people in poverty. Drawing upon a survey of people on low incomes, the charity proposing.

• Working with people on low incomes to understand the hidden costs in different health care settings.
• Collate best practice, develop support guidelines and share across health care settings.
• Raise awareness amongst staff of the causes and consequences of living in poverty and services available.

## Ethics statement

Not required.

## Availability of data and materials

The data supporting this article's conclusions are available in the referenced studies.

## Authors' contributions

JF conceptualized the study. AGh, AGk and DL conducted the literature review and data extraction. AGh, EC, AGk, LL and PT supported the interpretation of findings AGH led the manuscript writing. All authors contributed to and approved the final manuscript.

## Disclosure statement

This report is independent research supported by the National Institute for Health and Care Research ARC North Thames and NHS England. The views expressed in this publication are those of the author(s) and not necessarily those of the National Institute for Health and Care Research, NHS England, or the Department of Health and Social Care.

## Funding

This review was commissioned by 10.13039/100030827NHS England.

## Declaration of competing interest

The authors declare the following financial interests/personal relationships which may be considered as potential competing interests:

This report is independent research supported by the National Institute for Health and Care Research ARC North Thames and NHS England. The views expressed in this publication are those of the author(s) and not necessarily those of the National Institute for Health and Care Research, NHS England, or the Department of Health and Social Care.
